# Supplementary material for: Integrated transcriptome study of the tumor microenvironment for treatment response prediction in male predominant hypopharyngeal carcinoma
Source: Nat Commun. 2023 Mar 16;14:1466. doi: 10.1038/s41467-023-37159-8 (PMC10020474; doi:10.1038/s41467-023-37159-8)
Supplement: Supplementary file 1 — Supplementary Information [file 41467_2023_37159_MOESM1_ESM.pdf]

Supplementary Information for

Integrated transcriptome study of the tumor microenvironment for treatment response prediction in male predominant hypopharyngeal carcinoma

Yang Zhang[1,#,*], Gan Liu[2,3,#,*], Min-Zhen Tao[2,#], Hui Ning[2], Wei Guo[1], Gao-Fei Yin[1], Wen Gao[1], Li-Fei Feng[1], Jin Gu[2], Zhen Xie[2,*], Zhi-Gang Huang[1,*]

1 Department of Otolaryngology Head and Neck Surgery, Beijing Tongren Hospital, Capital Medical University, Key Laboratory of Otolaryngology Head and Neck Surgery (Capital Medical University), Ministry of Education, Beijing, 100730, China.
2 MOE Key Laboratory of Bioinformatics and Bioinformatics Division, Center for Synthetic and System Biology, Department of Automation, Beijing National Research Center for Information Science and Technology, Tsinghua University, Beijing, 100084, China.
3 Tsinghua-Peking Joint Center for Life Sciences, Tsinghua University, 100084 Beijing, China.
# These authors contributed equally: Yang Zhang, Gan Liu, Min-Zhen Tao
* Corresponding authors: zhangyangent@163.com (Y.Z.)or wz_liugan@163.com (G.L.) or zhenxie@tsinghua.edu.cn (Z.X.) or huangzhigang1963@163.com (Z-G.H.)

Tables of Contents

Supplementary Figures 1-18

Supplementary Tables 1-5

Supplementary Figures

Supplementary Figure 1. Survival analysis between groups based on our HPC cohort, related to Figure 1

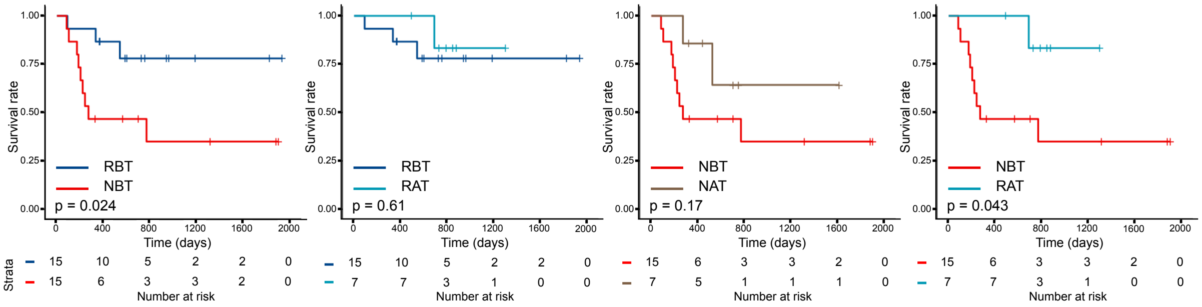

Supplementary Fig. 1 Survival analysis between groups based on our HPC cohort, related to Figure 1 Kaplan-Meier plots of survival analyses for patients in different groups in pairwise comparison. P-values were calculated by the log-rank test.

Supplementary Figure 2. The quality control filters in scRNA-seq data, related to Figure 1

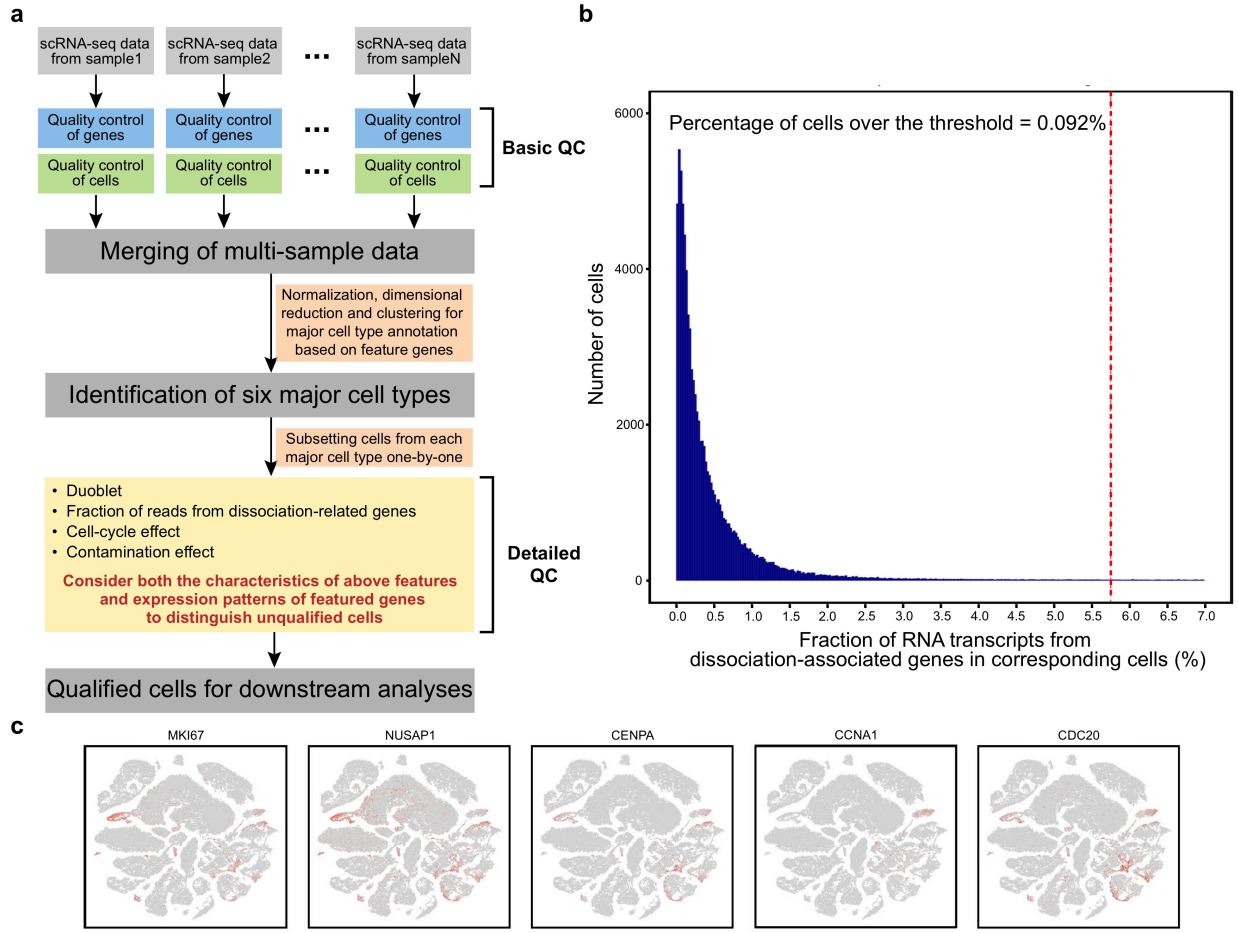

Supplementary Fig. 2 The quality control filters in scRNA-seq data, related to Figure 1

a Pipeline for quality control (QC) filters in scRNA-seq data to get qualified cells for downstream analyses.

b Low fraction of transcripts expressing dissociation-associated genes in cells after whole quality control.

c Quite small proportion of T/NK cells and epithelial cells expressing proliferation-related genes.

Supplementary Figure 3. The single-cell landscape in TME of HPC, related to Figure 1

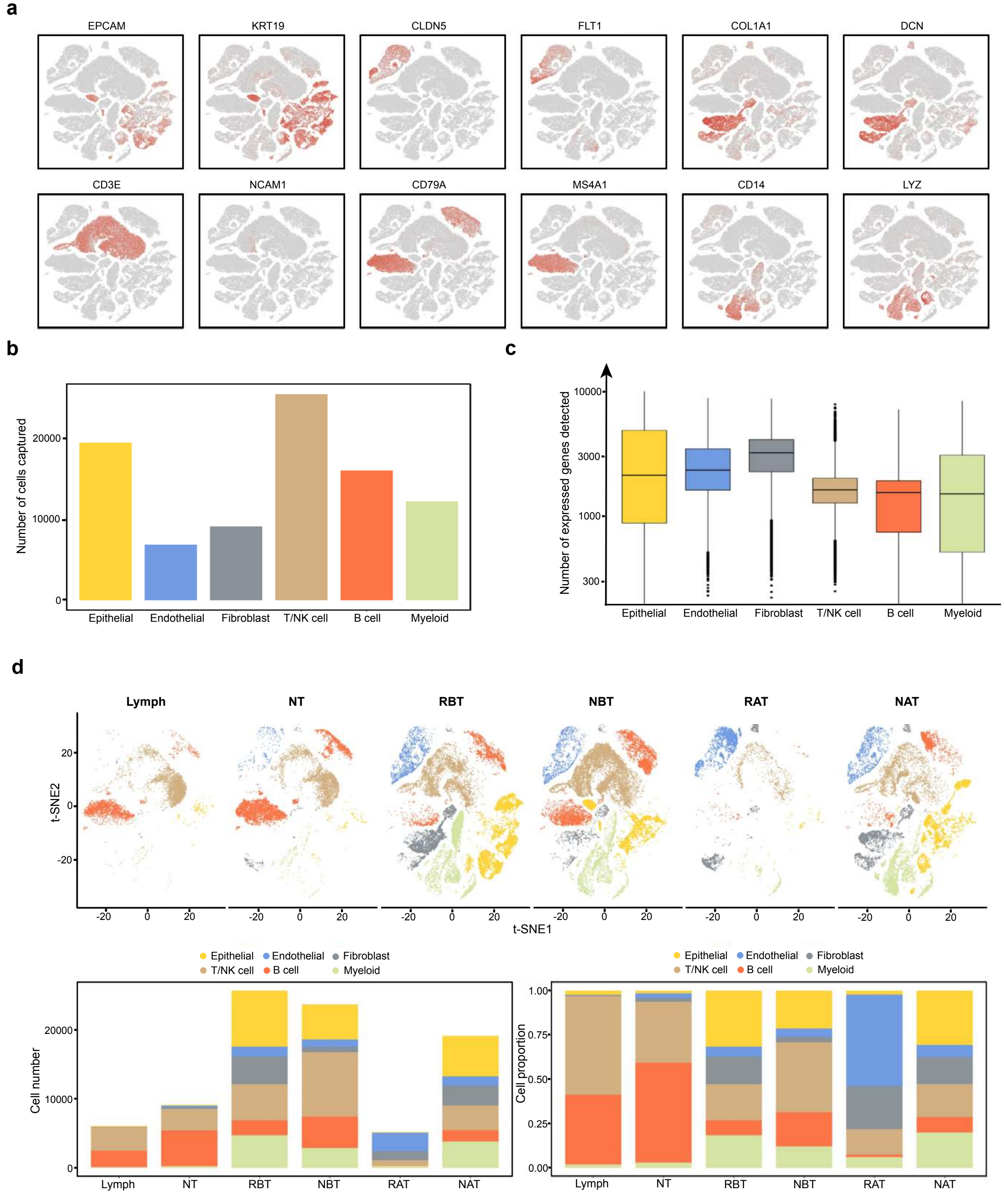

Supplementary Fig. 3 The single-cell landscape in TME of HPC, related to Figure 1

a t-SNE plots of extended canonical cell marker genes used to label six major cell types.

b Captured cell numbers of six major cell types. Source data are provided as a Source Data file.

c Numbers of expressed genes detected of six major cell types. The biological independent cell numbers of epithelial, endothelial, fibroblast, T/NK cell, B cell and myeloid were 19456, 6859, 9115, 25442, 16012 and

12210. The centers of box plots correspond to median values, with the boxes and whiskers corresponding to the corresponding interquartile ranges and 1.5× interquartile ranges; endpoints depict minimum and maximum values. Source data are provided as a Source Data file.
d t-SNE plots and statistical summaries for comparing the distribution of single cells among groups separated by radiological features and anatomical sites.

Supplementary Figure 4. The description of inter-tumor heterogenous malignant tumor cells, related to Figure 2

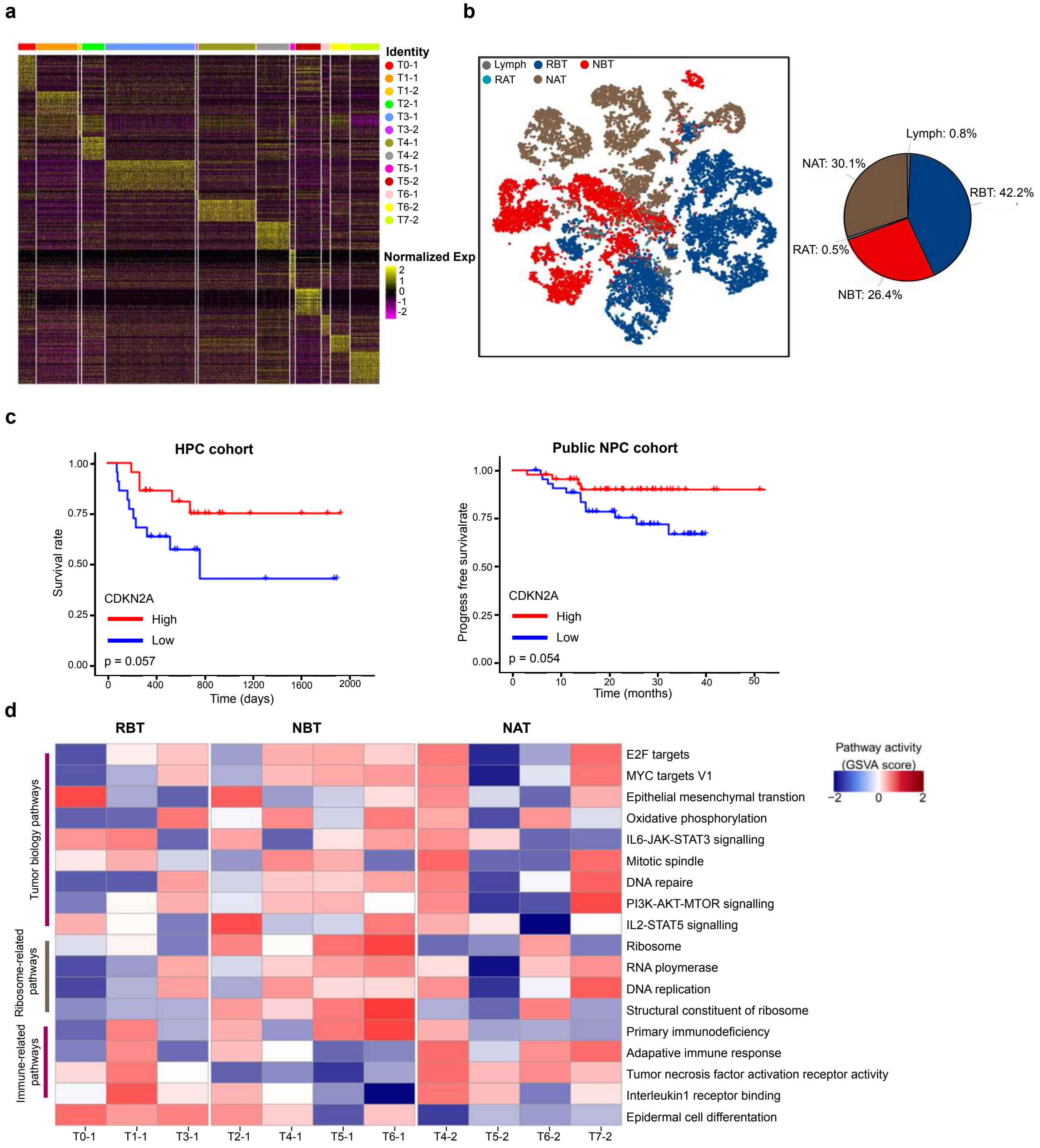

Supplementary Fig. 4 Description of inter-tumor heterogenous malignant tumor cells, related to Figure 2
a Heatmap showing differentially expressed genes across 13 individual samples.
b t-SNE plot of overall 19207 malignant tumor cells colored by annotation groups (left panel). Pie plot showing percentages of cells from each group (right panel).
c Kaplan-Meier plots analyzed from our HPC cohort with 44 patients (left panel) and public NPC cohort with 88 patients (right panel). The stratified high and low groups were divided by median gene expression levels in both plots. P-values were calculated by log-rank test.

d Heatmap showing different enrichments in tumor biology, RNA related, immune related signaling pathways across different samples from RBT, NBT and NAT groups. Source data are provided as a Source Data file.

Supplementary Figure 5. Characterization of functional gene modules from malignant tumor cells, related to Figure 2

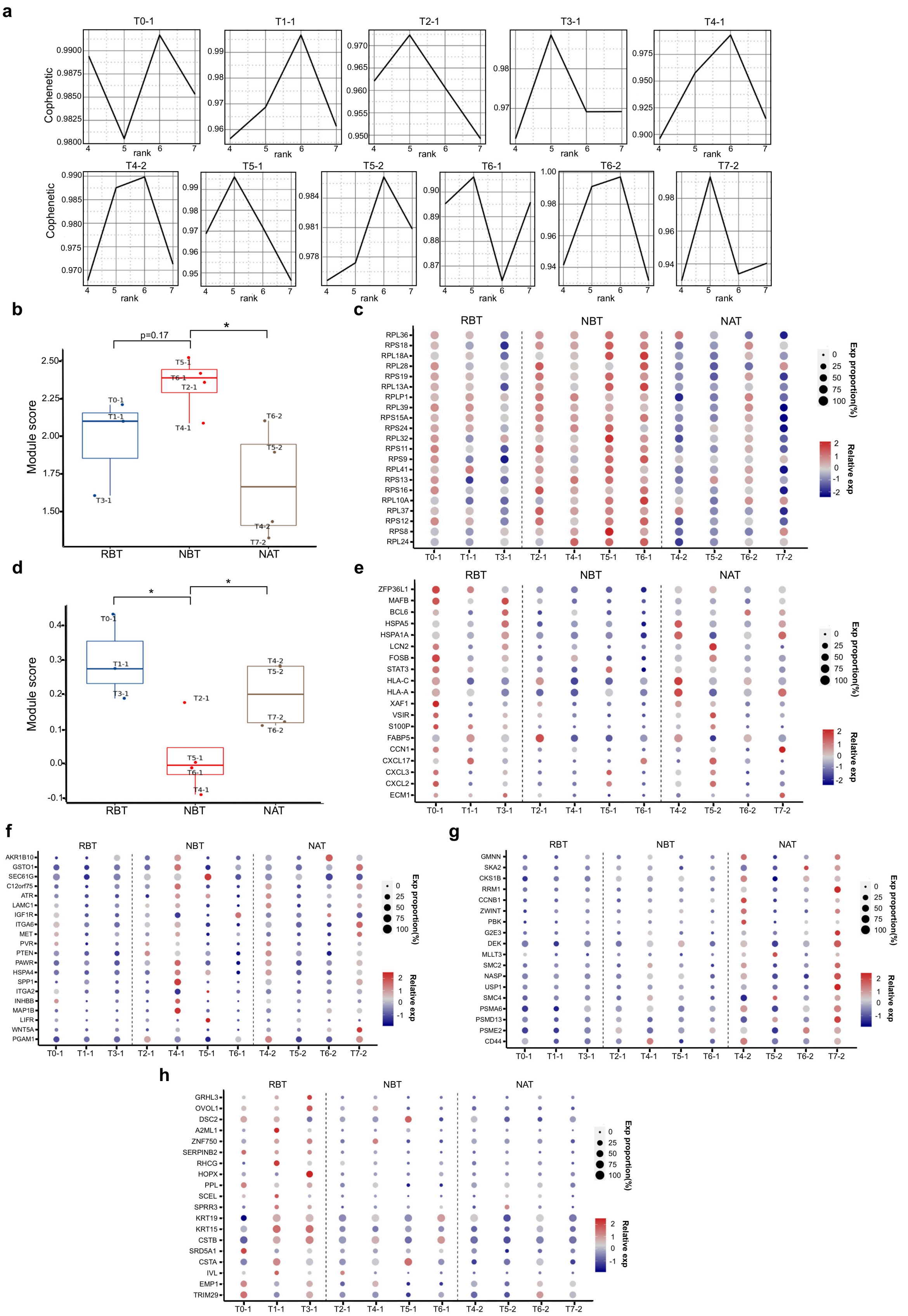

Supplementary Fig. 5 Characterization of functional gene modules from malignant tumor cells, related to Figure 2

a Pre-test for factorization parameter selection of each sample in NMF.

b Boxplot of module score in each sample across RBT (n = 3 biologically independent samples), NBT (n = 4 biologically independent samples) and NAT (n = 4 biologically independent samples) groups for Ribosome module using scRNA-seq data. P-values were calculated by two-sided Student's t test, and p-values ≤0.05 were represented as *. The centers of box plots correspond to median values, with the boxes and whiskers corresponding to the corresponding interquartile ranges and 1.5× interquartile ranges; colored dots denote each sample.

c Expression profiles of malignant tumor cells for genes from Ribosome module across different samples in RBT, NBT and NAT groups, separated by dashed vertical lines.

d Boxplot of module score in each sample across RBT (n = 3 biologically independent samples), NBT (n = 4 biologically independent samples) and NAT (n = 4 biologically independent samples) groups for Immunity module using scRNA-seq data. P-values were calculated by two-sided Student's t test, and p-values ≤0.05 were represented as *. The centers of box plots correspond to median values, with the boxes and whiskers corresponding to the corresponding interquartile ranges and 1.5× interquartile ranges; colored dots denote each sample.

e Expression profiles of malignant tumor cells for genes from Immunity module across different samples in RBT, NBT and NAT groups, separated by dashed vertical lines.

f Expression profiles of malignant tumor cells for genes from EMT_extended module across different samples in RBT, NBT and NAT groups, separated by dashed vertical lines.

g Expression profiles of malignant tumor cells for genes from Cell-cycle module across different samples in RBT, NBT and NAT groups, separated by dashed vertical lines.

h Expression profiles of malignant tumor cells for genes from Epi_development module across different samples in RBT, NBT and NAT groups, separated by dashed vertical lines.

All plots are provided with Source data in a Source Data file.

Supplementary Figure 6. Prognosis of three functional gene modules, related to Figure 2

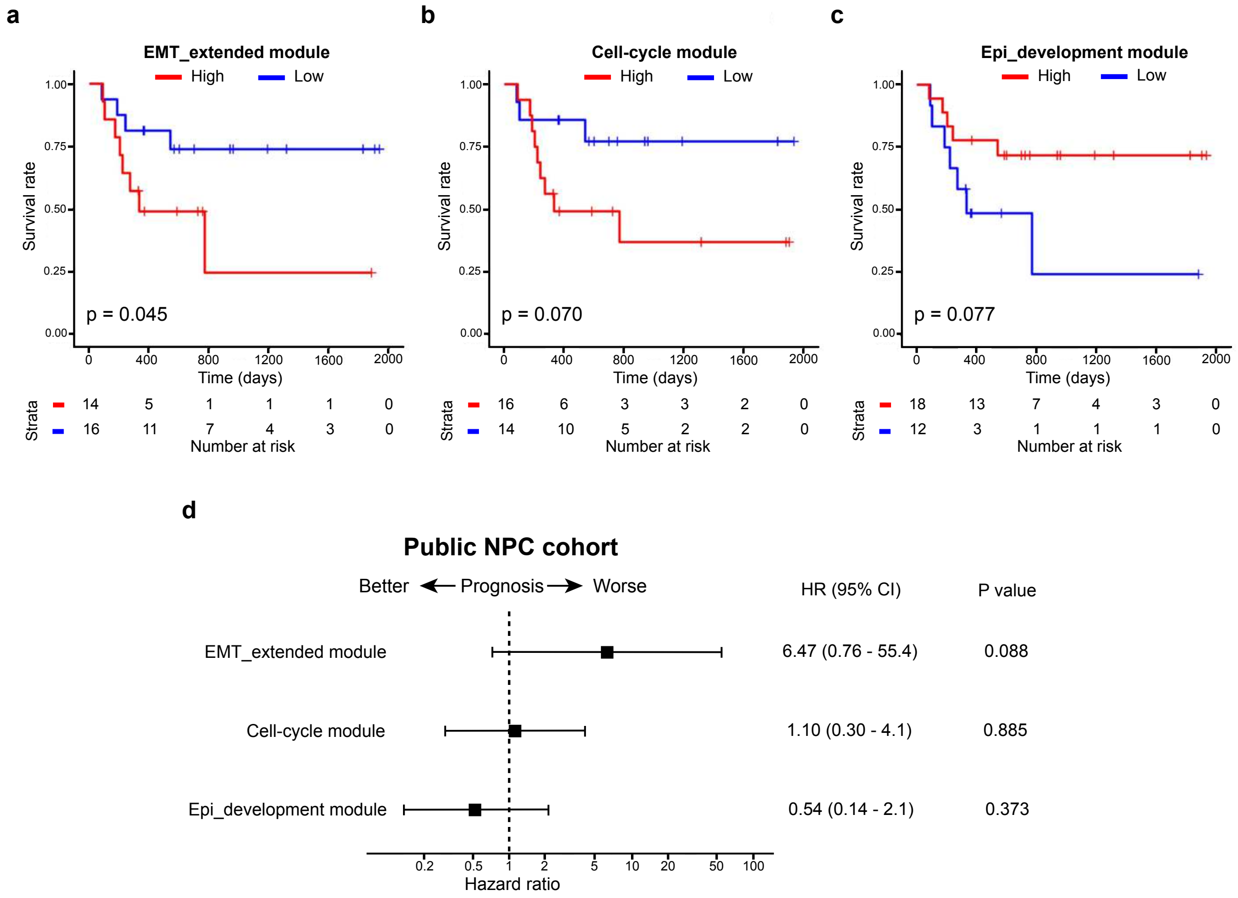

Supplementary Fig. 6 Prognosis of three functional gene modules, related to Figure 2

a-c Survival analyses of 30 patients from treatment-naïve in our HPC cohort with high or low expression scores of EMT_extended module (**a**), Cell-cycle module (**b**) and Epi_development module (**c**) separately. Patients were stratified by the median module scores. P-values were calculated by log-rank test.

d Prognostic values of EMT_extended, cell_cycle and epi_development modules in public NPC cohort (n = 88 biologically independent samples). Forest plot showed HRs (central black squares) and 95% CIs (horizontal ranges) derived from Cox regression survival analyses for progression-free survival in multivariable analyses adjusted for age, sex, smoking history and disease stage. Corresponding Cox regression p-values are also shown. CI, confidence interval.

Supplementary Figure 7. Detailed characterization of CD8+ T cells and NK cells, related to Figure 3

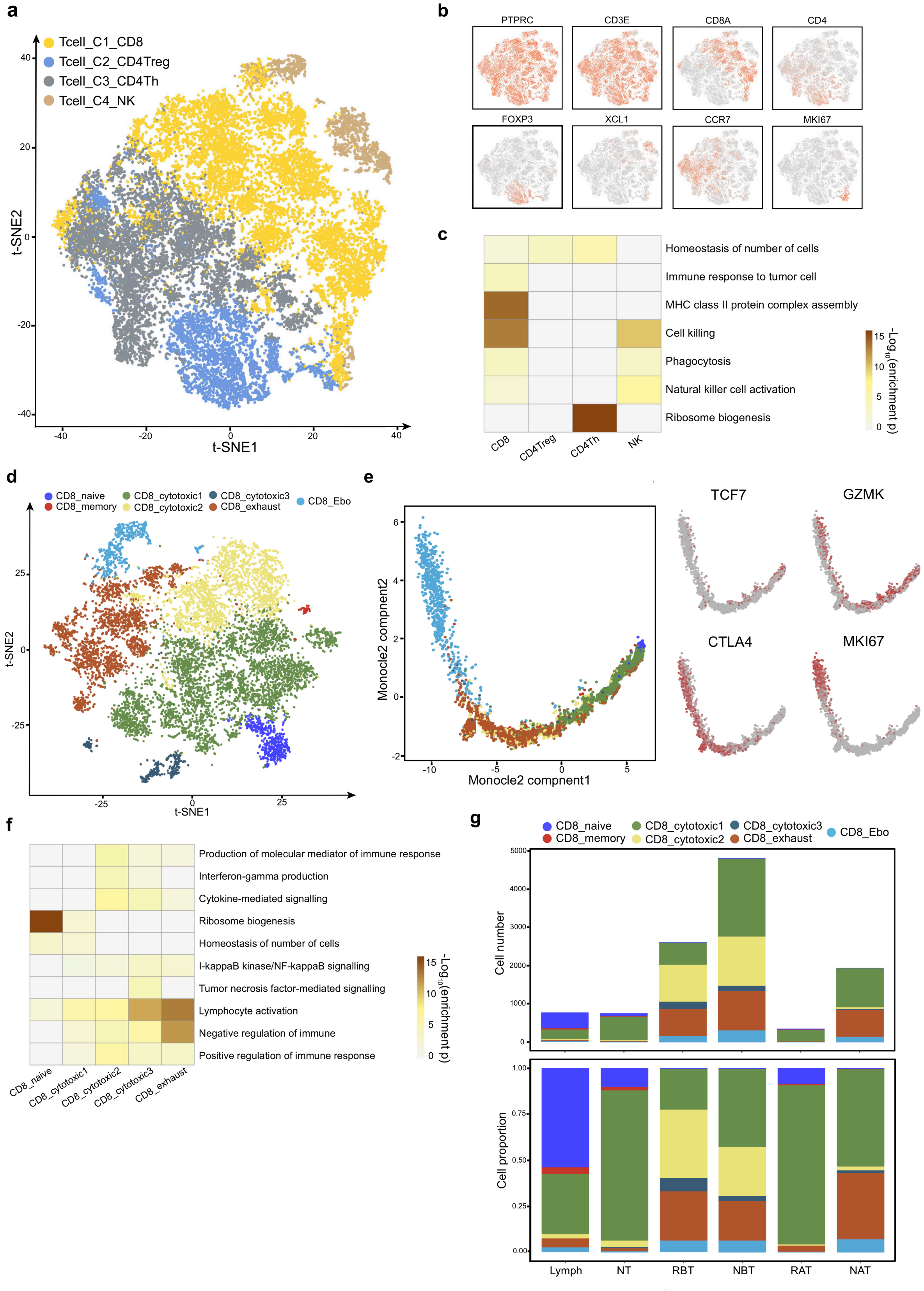

Supplementary Fig. 7 Detailed characterization of CD8+ T cells and NK cells, related to Figure 3

a Identification of NK cells and subclustering CD3E+ T cells into CD8+ T cells, CD4+ Th cells and CD4+ FOXP3+ Treg cells.

b Normalized expressions of canonical marker genes for T and NK cells. The depth of color from grey to red represents low to high expression.

c Heatmap showing the selected signalling pathways (rows) with significant enrichment of GO and KEGG terms for CD8+ T cells, Th, Treg and NK cells. Source data are provided as a Source Data file.

d t-SNE plot of CD8+ T cells annotated into seven subtypes. Each dot represents one single cell, colored according to cell subtype.

e Pseudotime developmental trajectory analysis of CD8+ T cells with representative marker genes' expression. Each dot represents one single cell, colored according to cell subtype.

f Heatmap showing the selected signalling pathways (rows) with significant enrichment of GO and KEGG terms for five CD8+ T cell subtypes. Source data are provided as a Source Data file.

g Cell numbers and cell proportion differences of seven CD8+ T subtypes among different groups. Source data are provided as a Source Data file.

Supplementary Figure 8. Detailed characterization of CD4+ T cells, related to Figure 3

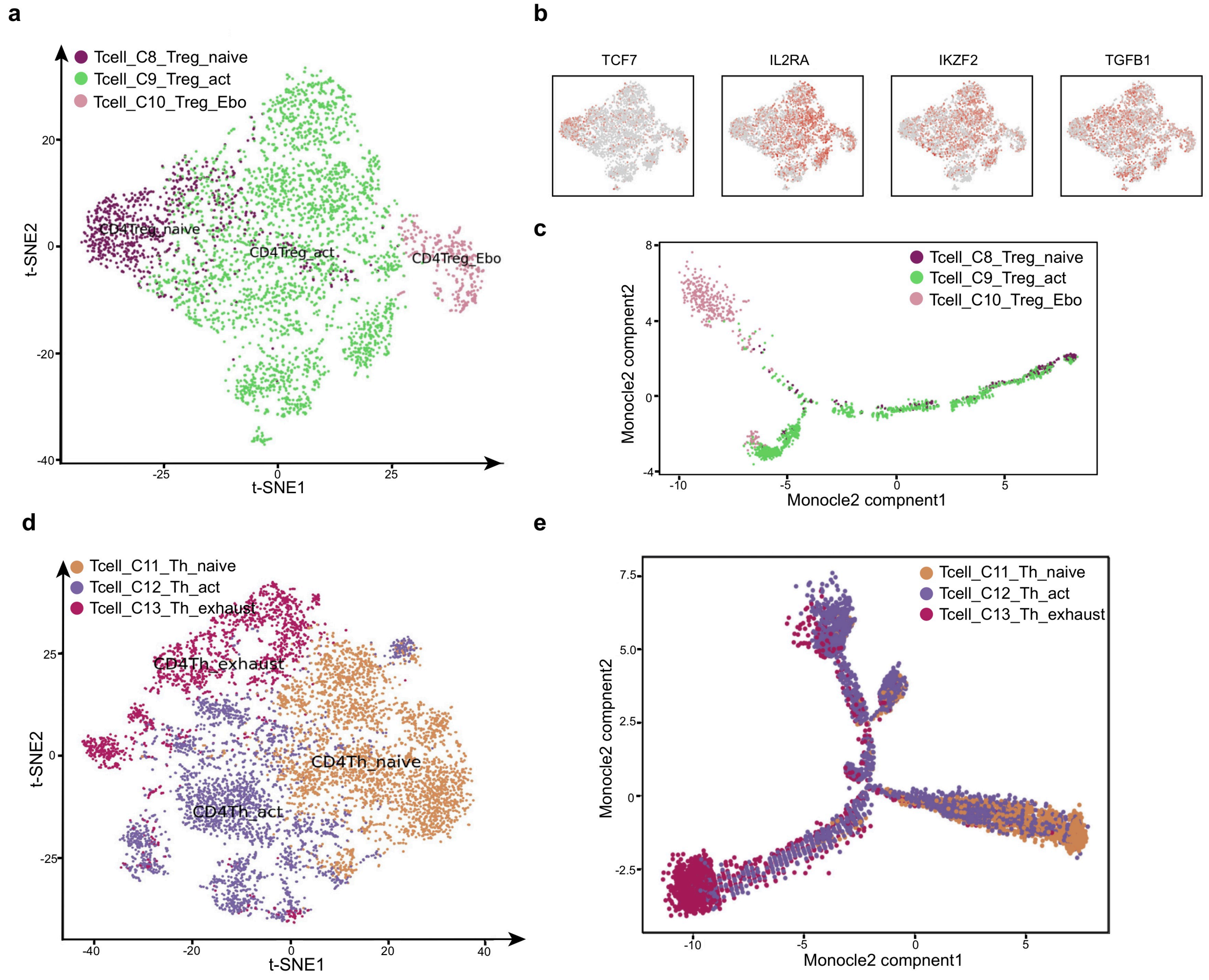

Supplementary Fig. 8 Detailed characterization of CD4+ T cells, related to Figure 3

a t-SNE plot of CD4+ FOXP3+ Treg cells annotated into three subtypes. Each dot represents one single cell, colored according to cell subtype.

b Normalized expressions of canonical marker genes for each subtypes of Treg cells. The depth of color from grey to red represents low to high expression.

c Pseudotime developmental trajectory analysis of Treg cells with representative marker genes' expression. Each dot represents one single cell, colored according to cell subtype.

d t-SNE plot of CD4+ Th cells annotated into three subtypes. Each dot representing one single cell, colored according to cell subtype.

e Pseudotime developmental trajectory analysis of Th cells with representative genes marker genes' expression. Each dot represents one single cell, colored according to cell subtype.

Supplementary Figure 9. Detailed characterization of myeloid cells, related to Figure 3

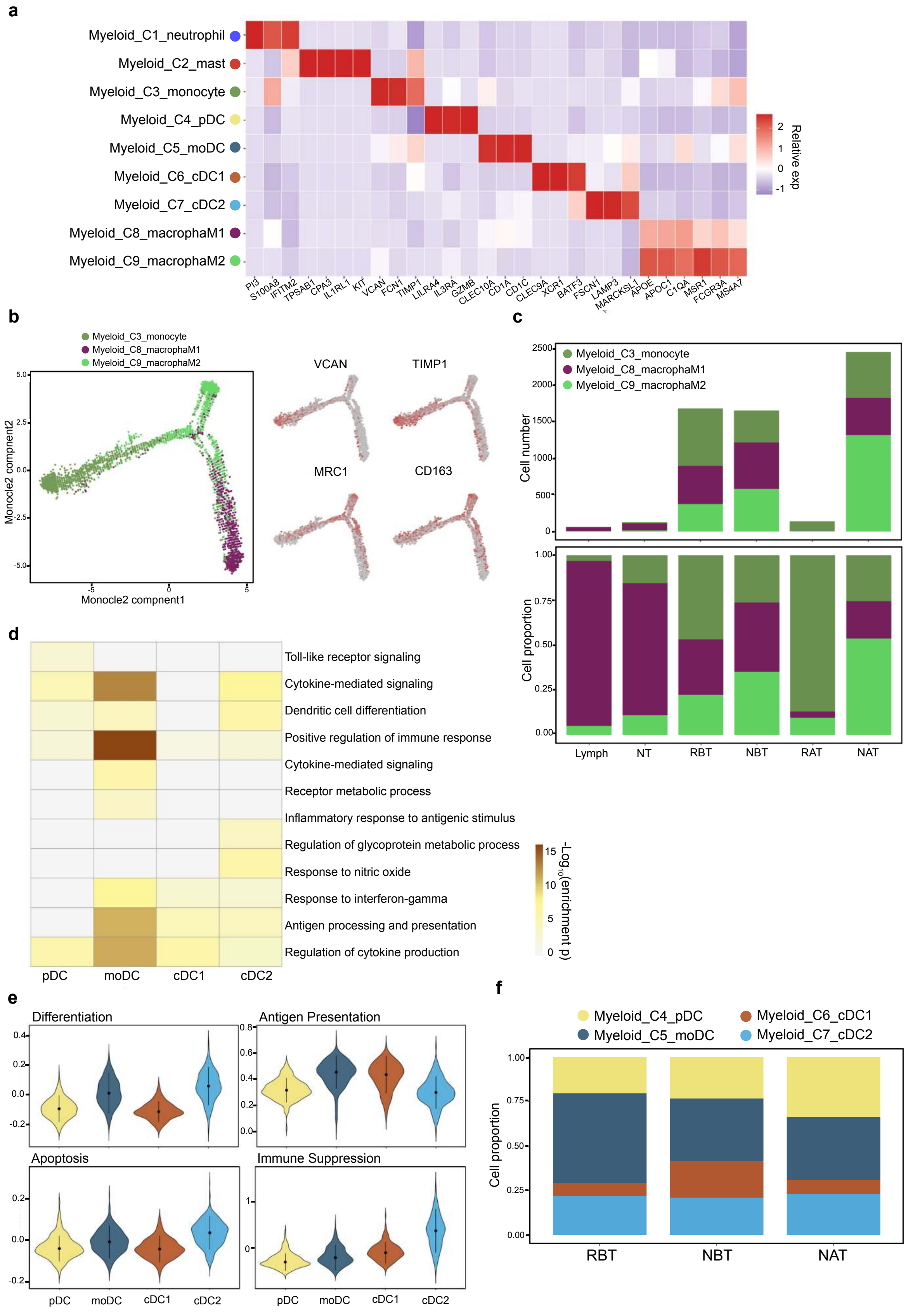

Supplementary Fig. 9 Detailed characterization of myeloid cells, related to Figure 3

a Heatmap showing average gene expressions for nine myeloid cell subtypes, including neutrophil, mast, monocyte, four DC subtypes and two macrophage subtypes. Source data are provided as a Source Data file.

b Pseudotime developmental trajectory analysis of monocyte and macrophage cells with representative genes marker genes' expression. Each dot represents one single cell, colored according to cell subtype.

c Cell numbers and cell proportion differences of three monocyte and macrophage subtypes among different groups. Source data are provided as a Source Data file.

d Heatmap showing the selected signalling pathways (rows) with significant enrichment of GO and KEGG terms for four DC subtypes (pDC, moDC, cDC1, cDC2). Source data are provided as a Source Data file.

e Violin plots showing the expression scores of differentiation, apoptosis, antigen presentation and immune suppression signature gene-sets for four DC subtypes (n=1455). Inside black points denote median values and lines denote the corresponding interquartile ranges.Source data are provided as a Source Data file.

f Cell numbers and cell proportion differences of four DC subtypes among RBT, NBT and NAT groups. Source data are provided as a Source Data file.

Supplementary Figure 10. Detailed characterization of B cells, related to Figure 3

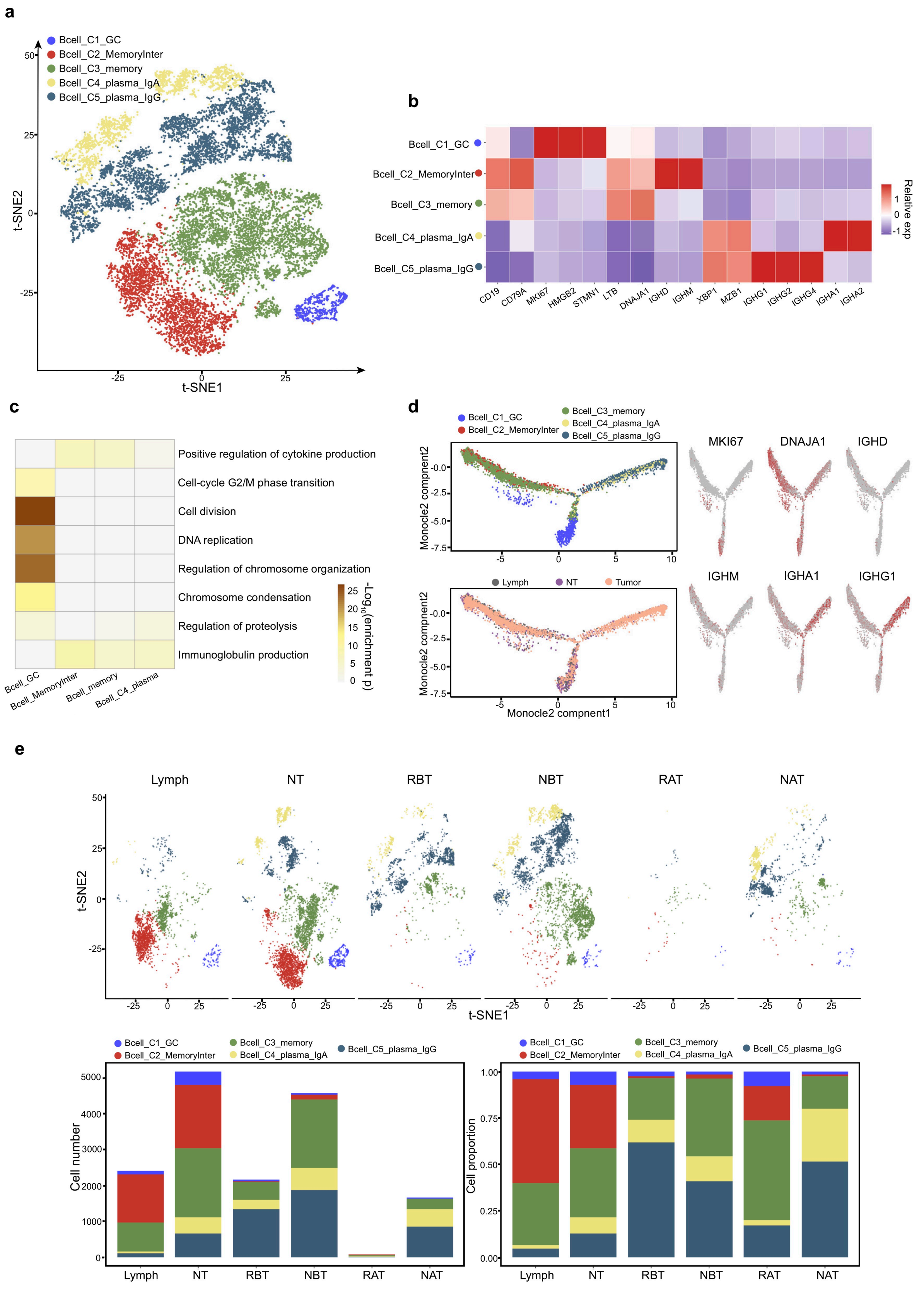

Supplementary Fig. 10 Detailed characterization of B cells, related to Figure 3

a t-SNE plot of B cells annotated into five subtypes. Each dot represents one single cell, colored according to cell subtype.

b Heatmap showing average gene expressions for five B cell subtypes. Source data are provided as a Source Data file.

c Heatmap showing the selected signalling pathways (rows) with significant enrichment of GO and KEGG terms for B cell subtypes (B_GC, B_MemoInter, B_Memory, B_Plasma). Source data are provided as a Source Data file.

d Pseudotime developmental trajectory analysis of B cells with representative genes marker genes' expression. Each dot represents one single cell, colored according to cell subtype.

e Cell numbers and cell proportion differences of five B cell subtypes among different groups. Source data are provided as a Source Data file.

Supplementary Figure 11. Detailed characterization of endothelial cells, related to Figure 3

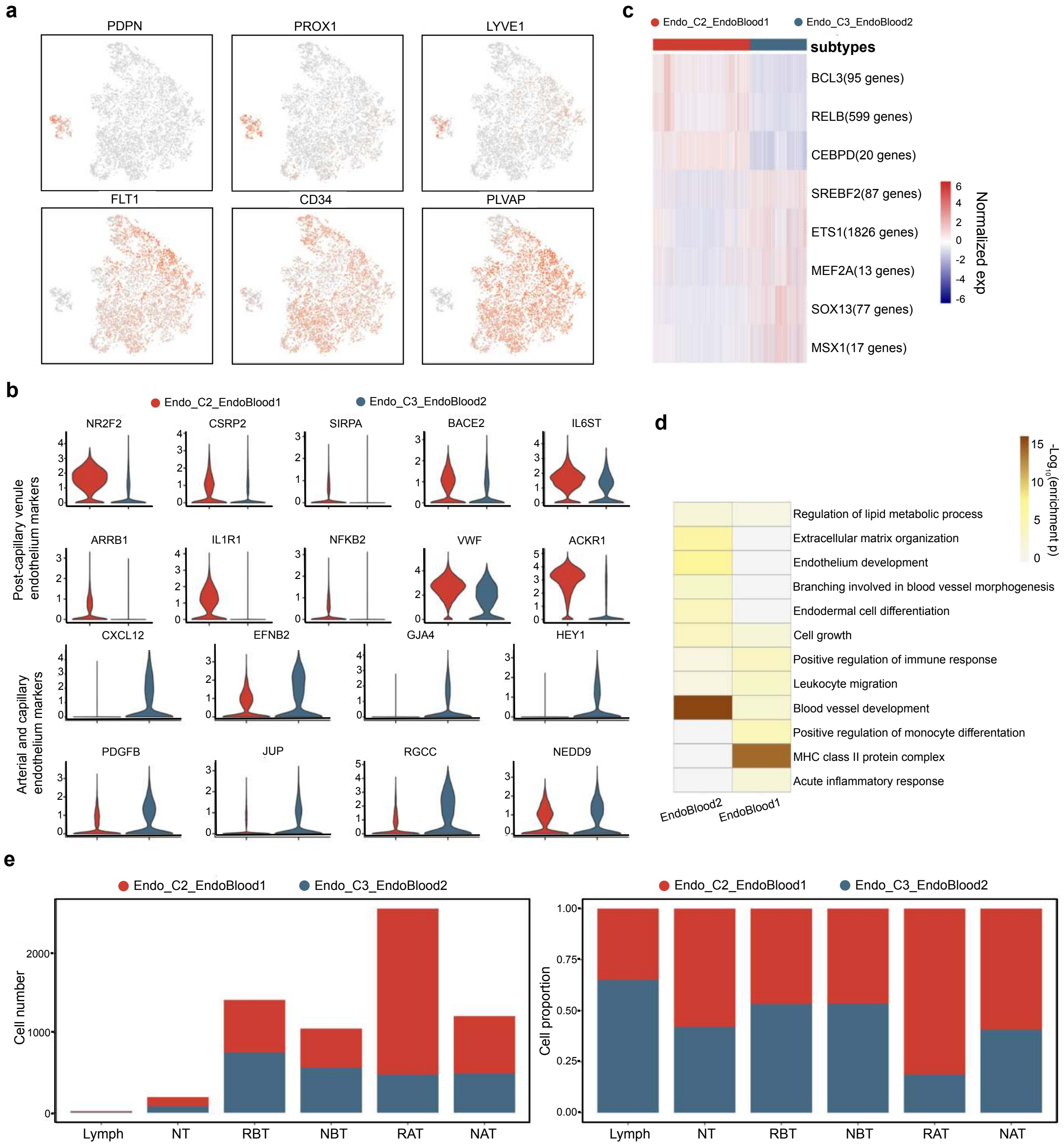

Supplementary Fig. 11 Detailed characterization of endothelial cells, related to Figure 4

a Normalized expressions of canonical marker genes to distinguish lymphatic and vascular endothelium. The depth of color from grey to red representing low to high expression.

b Violin plots showing the expression levels of post-capillary venule endothelium and arterial and capillary endothelium markers in subtypes of Endoblood1 and Endoblood2.

c Heatmap showing the activity of TF regulons for Endoblood1 and Endoblood2.

d Heatmap showing the selected signalling pathways (rows) with significant enrichment of GO and KEGG terms for Endoblood1 and Endoblood2. Source data are provided as a Source Data file.

e Cell number and cell proportion differences of two vascular endothelium subtypes among groups. Source data are provided as a Source Data file.

Supplementary Figure 12. Detailed characterization of fibroblasts, related to Figure 4

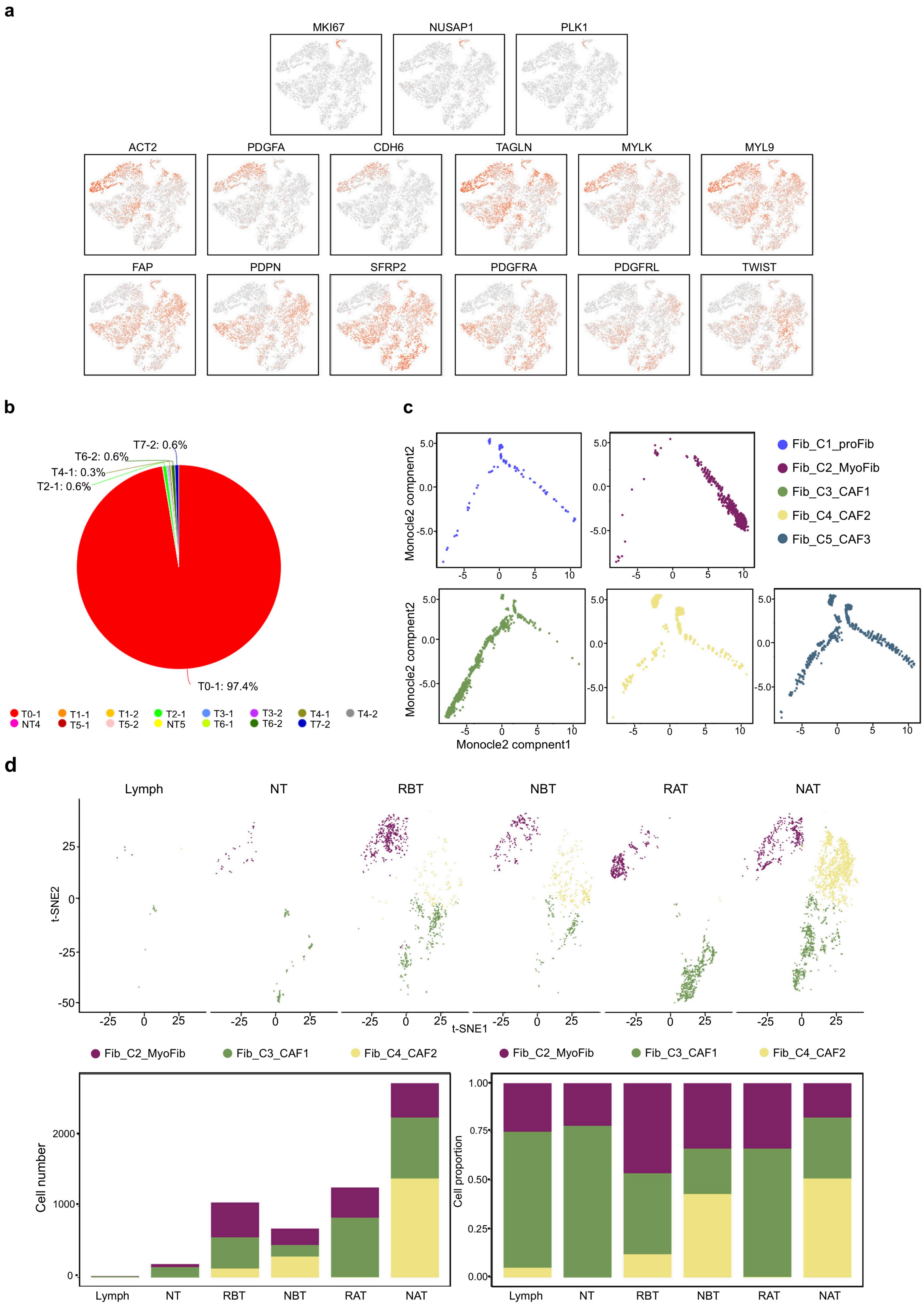

Supplementary Fig. 12 Detailed characterization of fibroblasts, related to Figure 4
a Normalized expressions of canonical marker genes to distinguish different fibroblast subtypes. The depth of color from grey to red representing low to high expression.
b The pie plot showing the sample origins of CAF3.
c Pseudotime developmental trajectory analysis of fibroblasts. Each dot representing one single cell, colored according to cell subtype.
d t-SNE plots and statistical summaries for comparing the distribution of single cells derived from different groups, separated by radiological features and anatomical sites. Source data are provided as a Source Data file.

Supplementary Figure 13. Comparison of intercellular interactions among three groups, related to Figure 5

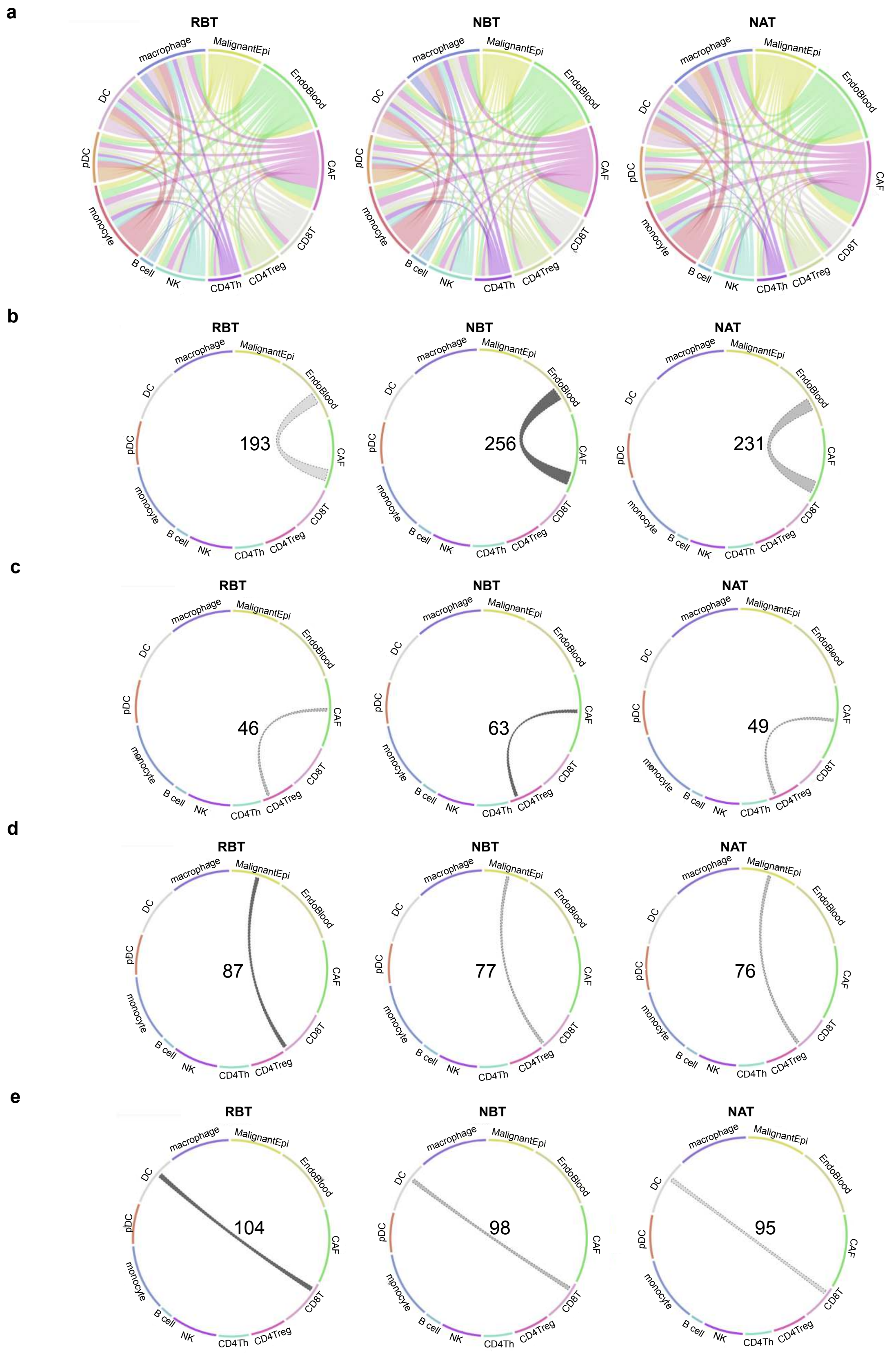

Supplementary Fig. 13 Comparison of intercellular interactions among three groups, related to Figure5
a Circos plots showing the overall intercellular interactions among different cell types in RBT, NBT and NAT groups. The strings are non-directional and represent interactions determined on the basis of expression of a ligand by one cell type and expression of a corresponding receptor by another cell type. The thickness of each string corresponds to the links of different interaction pairs, colored according to cell type.
b Numbers of cellular interactions between EndoBlood and CAF among RBT, NBT and NAT groups.
c Numbers of cellular interactions between CAF and Treg among RBT, NBT and NAT groups.
d Numbers of cellular interactions between malignant epithelial tumor cells and CD8+ T cells among RBT, NBT and NAT groups.
e Numbers of cellular interactions between CD8+ T cells and DCs among RBT, NBT and NAT groups.

Supplementary Figure 14. Pre-test of input cell subtypes for CIBERSORTx, related to Figure 6

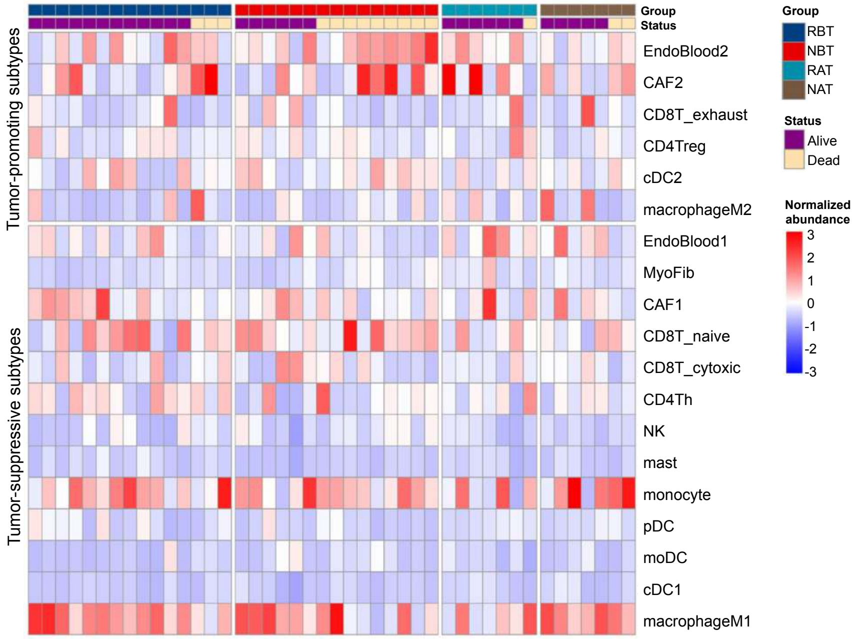

Supplementary Fig. 14 Pre-test of input cell subtypes for CIBERSORTx, related to Figure 6
Heatmap of the normalized cell abundance with nineteen subtypes estimated via CIBERSORTx and clinical records in our HPC cohort. All 44 samples from RBT, NBT, RAT and NAT groups were deconvolved for estimation. Source data are provided as a Source Data file.

Supplementary Figure 15. Correlations of subtype-specific signatures with clinical survival in HPC, related to Figure 6

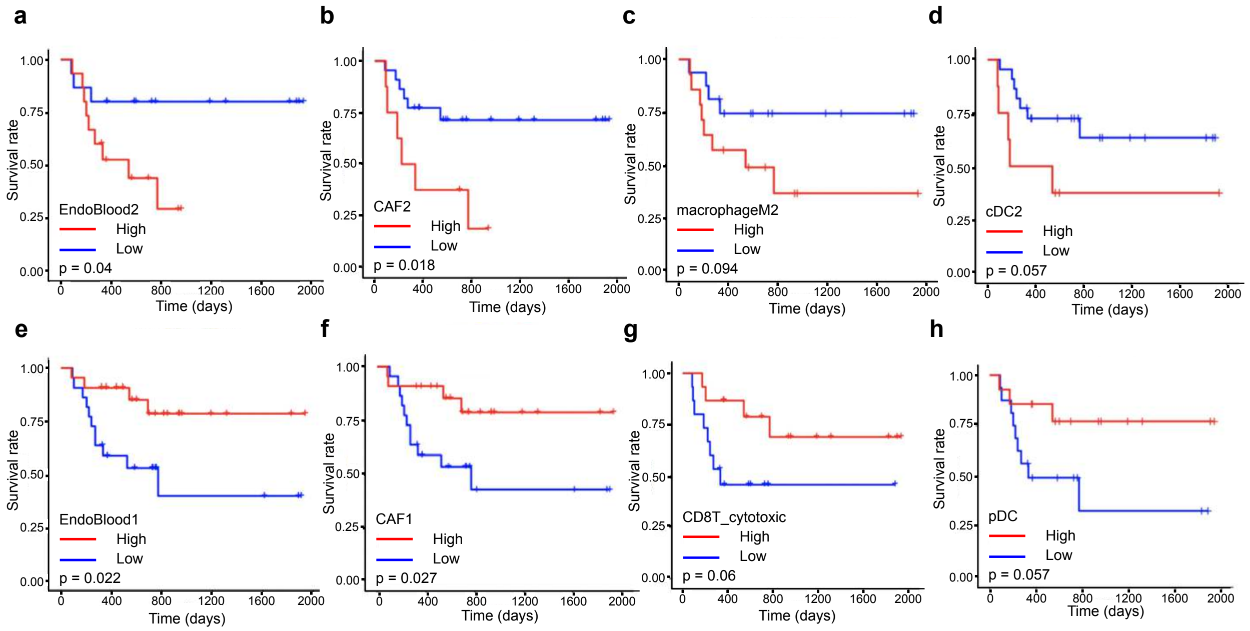

Supplementary Fig. 15 Correlations of subtype-specific signatures with clinical survival in HPC, related to Figure 6

a Survival analysis of 30 patients from treatment-naïve in our HPC cohort with high or low expression scores of the featured gene-set generated from EndoBlood2. Patients were stratified by the median score.

b Survival analysis of 30 patients from treatment-naïve in our HPC cohort with high or low expression scores of the featured gene-set generated from CAF2. The high and low groups were divided by the 25% quantile value of featured scores.

c Survival analysis of 30 patients from treatment-naïve in our HPC cohort with high or low expression scores of the featured gene-set generated from macrophageM2. Patients were stratified by the mean score.

d Survival analysis of 30 patients from treatment-naïve in our HPC cohort with high or low expression scores of the featured gene-set generated from cDC2. The high and low groups were divided by the 25% quantile value of featured scores.

e Survival analysis of 44 patients in our HPC cohort with high or low expression scores of the featured gene-set generated from EndoBlood1. Patients were stratified by the median score.

f Survival analysis of 44 patients in our HPC cohort with high or low expression scores of the featured gene-set generated from CAF1. Patients were stratified by the median score.

g Survival analysis of 30 patients from treatment-naïve in our HPC cohort with high or low expression scores of the featured gene-set generated from CD8T_cytotoxic. Patients were stratified by the median score.

h Survival analysis of 30 patients from treatment-naïve in our HPC cohort with high or low expression scores of the featured gene-set generated from pDC. Patients were stratified by the mean score.

P-values in all plots above were calculated by the log-rank test.

Supplementary Figure 16. Potential clinical application of the prediction model, related to Figure 6

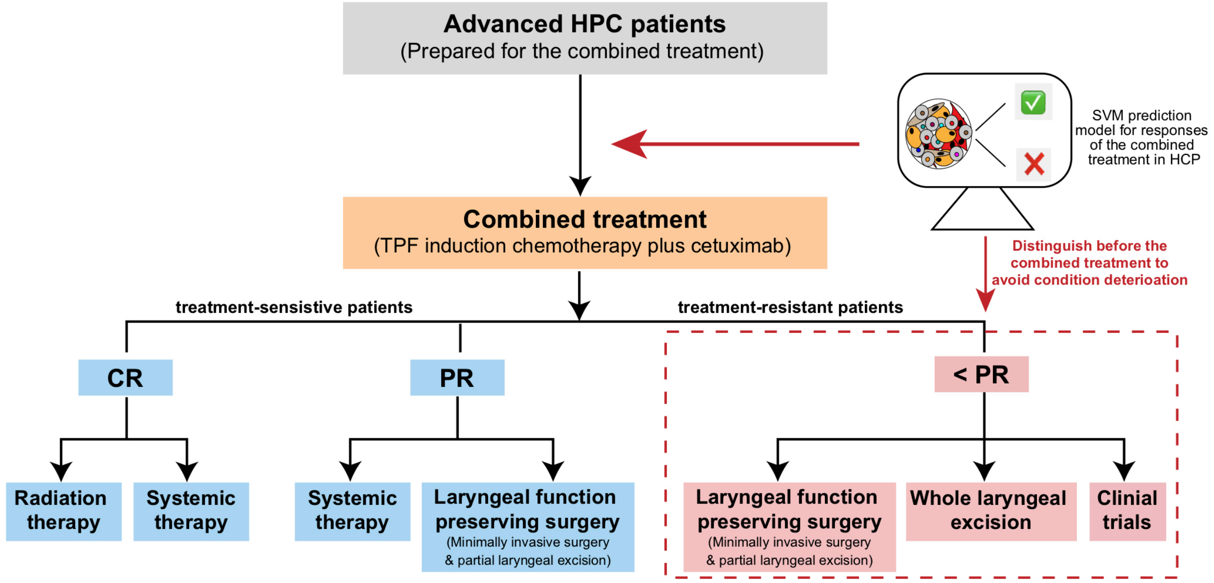

Supplementary Fig. 16 Potential clinical application of the prediction model, related to Figure 6
Patients accepted the combined treatment would come to three different conditions with radiological assessment, including complete response (CR), partial response (PR) and non-deceasing in tumor size (< PR). The first two kinds could be defined as treatment-sensitive groups, and the last one as treatment-resistant group. After the combined treatment, patients in the three groups would get corresponding further therapeutic strategies separately. Conditions of patients in the <PR group went deterioration after the ineffective combined treatment and had large possibility to receive whole laryngeal excision or be recommended for other clinical trials. With our established SVM prediction model, treatment-resistant patients would be distinguished in time to strive for available surgery or clinical trials to avoid deterioration.

Supplementary Figure 17. Extrapolation of the prediction method in NPC, related to Figure 6

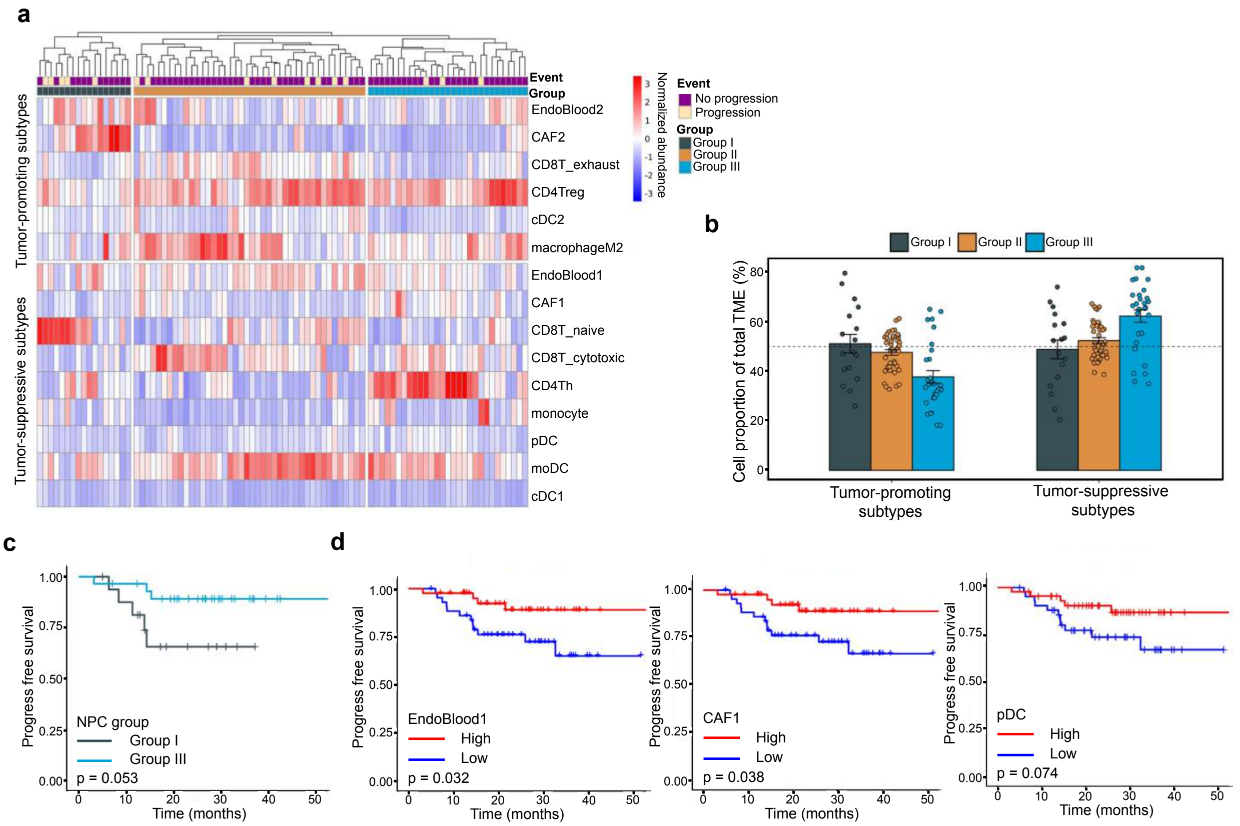

Supplementary Fig. 17 Extrapolation of the prediction method in NPC, related to Figure 6

a. Heatmap of the normalized cell abundance with fifteen subtypes estimated via CIBERSORTx and clinical records for 88 patients in public NPC cohort. Three subgroups were grouped under hierarchical clustering. Source data are provided as a Source Data file.

b. Average cell compositions in TME among three clustered groups by dividing fifteen subtypes into two groups named as tumor-promoting subtypes and anti-tumor subtypes. Error bars representing standard errors of cell constitutions in corresponding groups. The sample numbers in Group I to III were 17, 42 and 29 respectively. Source data are provided as a Source Data file.

c. Kaplan-Meier plot of survival analysis for patients in clustered group I and group III. P-value was calculated by the log-rank test.

d. Survival analyses of 88 public NPC patients with high or low expression scores of featured gene-sets generated from cell subtypes including EndoBlood1, CAF1 and pDC. Patients were stratified by the median scores for EndoBlood1 and CAF2, and by mean score for pDC. P-values were calculated by the log-rank test.

Supplementary Figure 18. Top ranking drugs for specified groups, related to Figure 6

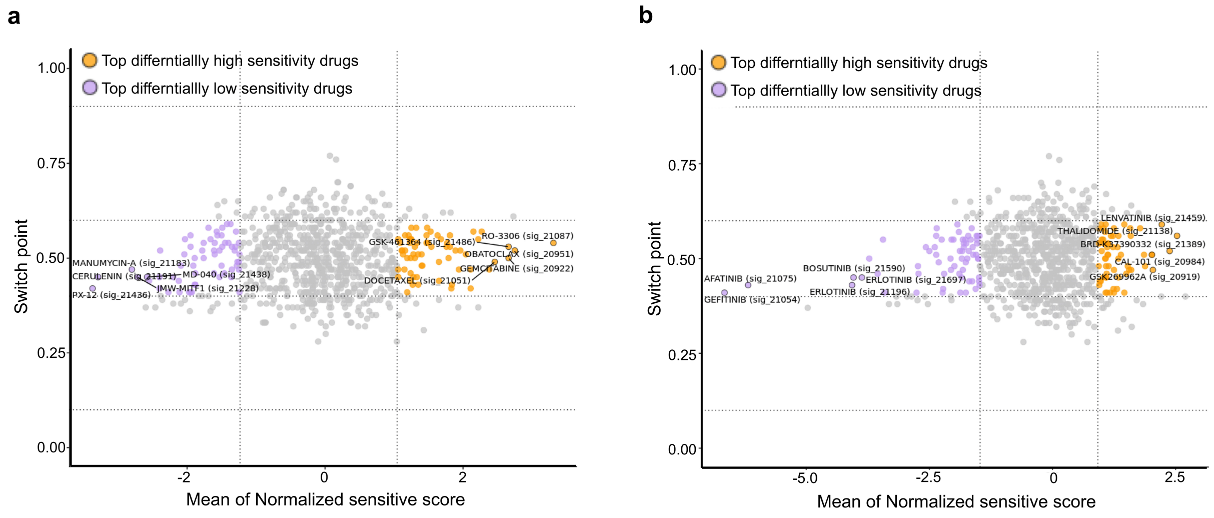

Supplementary Fig. 18 Top rank drugs for specific groups, related to Figure 6

a Scatter plot for drug ranking of malignant tumor cells in NBT group.

b Scatter plot for drug ranking of malignant tumor cells in NAT group.

Supplementary Tables

Supplementary Table 1. Clinical information of 44 HPC patients in our HPC cohort.

Patient ID	Group	Gender	Age	Smoke history (Yes/No)	Drinking history (Yes/No)	TMN stage	Time (Days)	Status
RBT1	RBT	male	68	Y	Y	T4N3M0	90	Dead
RBT2	RBT	female	67	N	N	T3N0M0	1936	Alive
RBT3	RBT	male	67	Y	Y	T3N0M0	959	Alive
RBT4	RBT	male	61	Y	N	T4N2M0	1827	Alive
RBT5	RBT	female	50	Y	Y	T3N0M0	938	Alive
RBT6	RBT	male	53	Y	Y	T4N2M0	756	Alive
RBT7	RBT	male	54	Y	N	T3N0M0	1189	Alive
RBT8	RBT	male	62	N	N	T2N2M0	584	Alive
RBT9	RBT	male	57	Y	Y	T4N2M0	724	Alive
RBT10	RBT	male	57	Y	Y	T4N2M0	333	Dead
RBT11	RBT	male	61	Y	Y	T3N2M0	600	Alive
RBT12	RBT	male	52	Y	Y	T4N2M0	541	Dead
RBT13	RBT	male	48	N	N	T3N2M0	361	Alive
RBT14	RBT	male	60	Y	N	T3N2M0	367	Alive
RBT15	RBT	male	48	N	N	T4N2M0	368	Alive
NBT1	NBT	male	57	Y	Y	T3N2M0	1903	Alive
NBT2	NBT	male	55	Y	Y	T4aN2cM0	83	Dead
NBT3	NBT	male	47	Y	N	T4N2M0	1882	Alive
NBT4	NBT	male	55	N	N	T4aN2bM0	1315	Alive
NBT5	NBT	male	65	Y	Y	T4aN1M0	172	Dead
NBT6	NBT	male	57	N	n	T4N2M0	241	Dead
NBT7	NBT	male	64	Y	Y	T4N2M0	699	Alive
NBT8	NBT	male	65	Y	Y	T2N2M1	185	Dead
NBT9	NBT	male	43	N	Y	T4N2M0	102	Dead
NBT10	NBT	male	69	Y	Y	T4N2M0	770	Dead
NBT11	NBT	male	49	Y	Y	T4N2M0	203	Dead
NBT12	NBT	male	44	Y	Y	T3NxM0	565	Alive
NBT13	NBT	male	62	Y	Y	T3N2M0	221	Dead
NBT14	NBT	male	57	Y	Y	T3N2M0	271	Dead
NBT15	NBT	male	61	Y	Y	T4N0M0	327	Alive
RAT1	RAT	male	57	Y	Y	T3N2M0	491	Alive
RAT2	RAT	male	57	Y	Y	T4N2M0	728	Alive
RAT3	RAT	male	46	Y	Y	T4N2M0	790	Alive
RAT4	RAT	male	71	Y	Y	T2N1M0	1299	Alive
RAT5	RAT	male	56	Y	Y	T2N2M0	689	Dead

RAT6	RAT	male	60	N	N	T3N1M0	846	Alive
RAT7	RAT	male	54	Y	N	T4N2M0	816	Alive
NAT1	NAT	male	51	Y	Y	T3N2M0	440	Alive
NAT2	NAT	male	55	N	N	T4N2M0	321	Alive
NAT3	NAT	male	57	Y	Y	T3N2M0	271	Dead
NAT4	NAT	male	64	Y	Y	T4N0M0	1613	Alive
NAT5	NAT	male	64	Y	Y	T4N2M0	699	Alive
NAT6	NAT	male	57	Y	Y	T4N2M0	747	Alive
NAT7	NAT	male	57	Y	Y	T4N2M0	524	Dead

Supplementary Table 2. Clinical information of HPC patients for 10x scRNA-seq

Patient ID	Gender	Age	Smoke history (Yes/No)	Drinking history (Yes/No)	TMN stage	Treatment response	Sample ID
P0	male	57	Y	Y	T4N2M0	sensitive	T0-1-CD45, T0-1-NonCD45
P1	male	57	Y	Y	T3N2M0	sensitive	T1-1, T1-1
P2	male	57	Y	Y	T3N2M0	resistant	T2-1
P3	male	62	Y	Y	T3N2M0	sensitive	T3-1, T3-2
P4	male	52	Y	Y	T4N2M0	resistant	T4-1, T4-2, NT4
P5	male	51	Y	Y	T3N2M0	resistant	T5-1, T5-2, NT5
P6	male	68	N	N	T2N2M0	resistant	T6-1, T6-2
P7	male	58	N	Y	T4N2M0	resistant	T7-2

We collected one sample from P0, and then split it into two parts by CD45 immunomagnetic beads and FACS before sending for library construction and scRNA-seq.

Supplementary Table 3. Characteristics of scRNA-seq samples

Sample ID	Group	raw events	Mean reads per event	Median gene per event	Qualified cells
T0-1-CD45	RBT	7253	53994	1503	11941
T0-1-NonCD45	RBT	7175	51111	2646	
T1-1	RBT	6145	69827	1005	4969
T1-2	Lymph	6933	54612	1565	6097
T2-1	NBT	6628	63957	1848	5299
T3-1	RBT	10569	33434	1657	8813
T3-2	RAT	6357	57329	1778	5228
T4-1	NBT	7828	43027	1197	6578
T4-2	NAT	8839	50913	1987	7589
NT4	NT	5677	60134	1621	4305
T5-1	NBT	6644	67327	1883	5744
T5-2	NAT	7284	64553	1654	1632
NT5	NT	5993	78710	1611	4863
T6-1	NBT	7145	46621	1420	6092
T6-2	NAT	6654	49849	1794	5432
T7-2	NAT	7529	51337	2025	4512

Supplementary Table 4. Gene-sets included in this study.

Description of gene set	Genes
**Ribosome module**	RPL36, RPS18, RPL18A, RPL28, RPS19, RPL13A, RPLP1, RPL39, RPS15A, RPS24, RPL32, RPS11, RPS9, RPL41, RPS13, RPS16, RPL10A, RPL37, RPS12, RPS8, RPL24
**Cell-cycle module**	GMNN, SKA2, CKS1B, RRM1, CCNB1, ZWINT, PBK, G2E3, DEK, MLLT3, SMC2, NASP, USP1, SMC4, PSMA6, PSMD13, PSME2, CD44
**Epi_development module**	GRHL3, OVOL1, DSC2, A2ML1, ZNF750, SERPINB2, RHCG, HOPX, PPL, SCEL, SPRR3, KRT19, KRT15, CSTB, SRD5A1, CSTA, IVL, EMP1, TRIM29
**EMT_extended module**	AKR1B10, GSTO1, SEC61G, C12orf75, ATR, LAMC1, IGF1R, ITGA6, MET, PVR, PTEN, PAWR, HSPA4, SPP1, ITGA2, INHBB, MAP1B, LIFR, WNT5A, PGAM1
**Immunity module**	ZFP36L1, MAFB, BCL6, HSPA5, HSPA1A, LCN2, FOSB, STAT3, HLA-C, HLA-A, XAF1, VSIR, S100P, FABP5, CCN1, CXCL17, CXCL3, CXCL2, ECM1
**Score calculating for CD8+ T naïve**	TCF7, SELL, LEF1, CCR7
**Score calculating for CD8+ T cytotoxic**	GZMA, GZMB, GZMH, GNLY, NKG7, PRF1, SLC4A10, S100A4, S100A6
**Score calculating for CD8+ T exhaustion**	CTLA4, PDCD1, HAVCR2, TIGIT, LAYN, TOX, BATF, ENTPD1, LAG3
**Score calculating for CD4+ Treg naïve**	TCF7, SELL, LEF1, CCR7
**Score calculating for CD4+ Tregstability**	FOXP3, IKZF2, IKZF4, IL2RA, IL2RB, IL2RG
**Score calculating for CD4+ Treg chemokines**	CCR8, CCR4, CXCR3, CXCR6, IL10, TGFB1
**Feature genes for survival analysis of Endoblood1**	CCL14, ACKR1, CLU, ADIRF, MMRN1, HLA-DRA, TGFBR3, IL1R1, NR2F2, HLA-DPB1, SELP, AQP1, TSPAN7, HLA-DRB1, PRCP, CD74, HLA-DPA1, IGFBP4, SNCG, RPL10, HLA-DQB1, ZNF385D, NPC2, SPARCL1, SELE, TGFBR2, IL33, LRATD2, TFPI, ZFP36L1, HLA-DMA, HLA-DRB5, LIFR, SDCBP, OLFM1, VWF, HLA-DQA1, PIM3, CAVIN2, POSTN, PDK4, TXNIP, CEBPD, CTSC, C7, ICAM1, IER3, DNASE1L3, VCAN, CYP1B1, CPE
**Feature genes for survival analysis of Endoblood2**	SEMA3G, NOTCH4, IGFBP3, HEY1, SLC9A3R2, GJA4, H19, GJA5, INSR, HES4, FBLN5, MMP2, RGCC, COL4A1, CXCL12, PODXL, ADAMTS6, LGALS1, HECW2, CALD1, BGN, PLCG2, ARL15, SRP14, DLL4, FN1, COL4A2, IL32, JAG2, MIR4435-2HG, ISG15, ADGRF5, CAVIN3, CYTOR, SAT1, RGS3, PLPP1, RHOB, ICAM2, IFI6, COL1A2, XAF1, DEPP1, FLT1, PMEPA1, S100A4, APCDD1, COL1A1, SOX17, JAG1, EFNB2, IGKC, IVNS1ABP, SRGN, CCN1, S100A8, S100A9, FABP4, ANGPT2, IGLC2, LTBP4, IGHA1
**Feature genes for survival analysis of CAF1**	CFD, CXCL14, APOD, CCDC80, OGN, CLU, IGF1, C3, SFRP2, TNXB, FBLN1, LTBP4, LEPR, SFRP1, ABI3BP, ABCA8, MGST1, ITM2A, PRELP, CST3, NFIA, LRP1, FGF7, CHRDL1, TSHZ2, AKR1C1, C1S, CYBRD1, PODN, EFEMP1, ABCA6, DCN, S100A6, PTGIS, SLPI, S100A10, CD34, NFIX, VIT, PLAC9, CELF2, PLA2G2A, IGFBP5, ELN, AKAP12, MGP, ANK2, ALDH1A1, METTL7A, ADD3, MFAP4, RARRES1, CXCL12, MT-ND3, NOVA1, TGFBR3, S100A13, PCOLCE2, SCARA5, SVEP1, MIR99AHG, NR2F1, IGFBP6, LIMA1, PI16, HELLPAR, ZFP36L2, SELENOP, LRRN4CL, ARL6IP5, SRPX, DCLK1, BOC, PDGFRA, FSTL1, GPNMB, RAMP2, MTRNR2L8, MEDAG, SERPINE2, ABLIM1, ANGPTL1, MTRNR2L12, IL6ST, MFAP5, FGL2, LTBP2, GSN, GPC3, MT-ND4L, SH3BP5, CP, DHRS3, OMD, USP53, FBLN5, SEMA3C, SPTBN1, SERPINA3, KLF4, CFH, COL14A1, IGFBP4, CYP1B1, CLEC3B, ITGBL1, ANXA1, F10, PLTP, PLPP1, CTSH, SSPN, PLPP3, TMEM176B, FCGRT, PIK3R1, C1QTNF3, OSR2, TMEM176A, LSP1, TXNIP, SFRP4, PTGDS, OAF, THBS4, MEG3, FBN1, PDGFRL, CD74, PLCG2, MT2A, CFB, ACKR3, CCN5, TIMP3, CRABP1, CD55, MT1X, IGKC, CCN2, SERPINE1
**Feature genes for survival analysis of CAF2**	MMP1, MMP11, POSTN, CTHRC1, MMP13, COL11A1, COL1A1, COL1A2, PLAU, COL3A1, COL7A1, LUM, FTH1, CTSK, COL10A1, HTRA1, IL7R, COL5A2, COL12A1, INHBA, MMP14, ADAMTS2, CRABP2, COL6A1, ISLR, WNT5A, COL5A1, RCN3, SLC16A3,

	TMEM158, CLEC11A, MFAP2, FAP, SPARC, WNT2, PDPN, RGS3, OSTC, THBS2, PTK7, ADAM12, SPHK1, CERCAM, SERPINH1, COL6A3, GREM1, GJA1, SDC1, FKBP10, PLPP4, GOLM1, CACNA2D3, TMEM45A, MYDGF, KDELR3, KDELR2, P4HB, EPSTI1, BMP1, SULF2, S100A16, PKM, BPGM, ARF4, PRDM1, PODNL1, PLAUR, COL27A1, PMEPA1, PPIB, AEBP1, COL6A2, SPON2, SULF1, LMCD1, TNFRSF12A, RCN1, TYMP, BASP1, HLA-B, STEAP1, ANTXR1, LY6E, CTSB, MRPS6, MMP2, EDIL3, RBP1, SSR3, PRDX4, ANGPTL2, ITGB5, IFI27, NREP, CALU, FMO1, GAS1, IFI6, DIO2, CXCL8, FTL, VCAN, MMP3, FN1, IL32, PRRX2, PCOLCE, BST2, RARRES2, OLFM2, COPZ2, TDO2, NEAT1, GBP1, ISG15, HSPA1A, EPYC, NBL1, ID1, MDK, IER3, TNFAIP6, ADM, CXCL5, ASPN, SAT1, CXCL1
**Feature genes for survival analysis of CD8T_cytotoxic**	GZMA, GZMB, GZMH, GNLY, NKG7, PRF1, SLC4A10, S100A4, S100A6
**Feature genes for survival analysis of pDC**	LILRA4, IL3RA, GZMB, TCF4, TCL1A, CLEC4C, JCHAIN, IRF4, MZB1
**Feature genes for survival analysis of cDC2**	FSCN1, LAMP3, MARCKSL1, SLCO5A1, CCL17, CD40
**Feature genes for survival analysis of macrophageM2**	ARG1, ARG2, IL10, FCGR2A, CD163, FCER2, CD200R1, PDCD1LG2, CD274, MARCO, CSF1R, MRC1, IL1RN, Il1R2, IL4R, CCL4, CCL13, CCL20, CCL17, CCL18, CCL22, CCL24, LYVE1, VEGFA, VEGFB, VEGFC, VEGFD, EGF, CTSA, CTSB, CSTC, CTSD, TGFB1, TGFB2, TGFB3, MMP14, MMP19, MMP9, CLEC7A, WNT7B, FASLG, TNFSF12, TNFSF8, CD276, VTCN1, MSR1, FN1, IRF4

Supplementary Table 5. Clinical information of addition 12 HPC patients for model test

Patient ID	Group	Gender	Age	Smoke history (Yes/No)	Drinking history (Yes/No)	TMN stage	Time (Days)	Status
addRBT1	RBT	male	61	Y	N	T3N0M0	1183	Alive
addRBT2	RBT	male	46	Y	Y	T4N2M0	749	Alive
addRBT3	RBT	male	62	Y	Y	T3N2M0	411	Alive
addRBT4	RBT	female	55	N	N	T4N0M0	570	Alive
addRBT5	RBT	male	54	Y	Y	T4N2M0	168	Alive
addRBT6	RBT	male	43	Y	Y	T3N3M0	223	Alive
addRBT7	RBT	male	46	Y	Y	T2N2M0	205	Alive
addNBT1	NBT	male	60	Y	Y	T3N1M0	698	Dead
addNBT2	NBT	male	62	Y	Y	T3N2M0	741	Alive
addNBT3	NBT	male	58	Y	Y	T4N2M0	178	Alive
addNBT4	NBT	male	73	Y	Y	T4N2M0	78	Alive
addNBT5	NBT	male	58	Y	Y	T4N2M0	338	Alive
