# Supplementary material for: Integrated transcriptome study of the tumor microenvironment for treatment response prediction in male predominant hypopharyngeal carcinoma
Source: Nat Commun. 2023 Mar 16;14:1466. doi: 10.1038/s41467-023-37159-8 (PMC10020474; doi:10.1038/s41467-023-37159-8)
Supplement: Supplementary file 3 — Reporting Summary [file 41467_2023_37159_MOESM3_ESM.pdf]

nature portfolio

Corresponding author(s): zhen xie

Last updated by author(s): Jan 31, 2023

Reporting Summary

Nature Portfolio wishes to improve the reproducibility of the work that we publish. This form provides structure for consistency and transparency in reporting. For further information on Nature Portfolio policies, see our Editorial Policies and the Editorial Policy Checklist.

Statistics

For all statistical analyses, confirm that the following items are present in the figure legend, table legend, main text, or Methods section.

n/a	Confirmed	
☐	☒	The exact sample size (*n*) for each experimental group/condition, given as a discrete number and unit of measurement
☐	☒	A statement on whether measurements were taken from distinct samples or whether the same sample was measured repeatedly
☐	☒	The statistical test(s) used AND whether they are one- or two-sided<br>*Only common tests should be described solely by name; describe more complex techniques in the Methods section.*
☐	☒	A description of all covariates tested
☐	☒	A description of any assumptions or corrections, such as tests of normality and adjustment for multiple comparisons
☐	☒	A full description of the statistical parameters including central tendency (e.g. means) or other basic estimates (e.g. regression coefficient) AND variation (e.g. standard deviation) or associated estimates of uncertainty (e.g. confidence intervals)
☐	☒	For null hypothesis testing, the test statistic (e.g. *F*, *t*, *r*) with confidence intervals, effect sizes, degrees of freedom and *P* value noted<br>*Give P values as exact values whenever suitable.*
☒	☐	For Bayesian analysis, information on the choice of priors and Markov chain Monte Carlo settings
☐	☒	For hierarchical and complex designs, identification of the appropriate level for tests and full reporting of outcomes
☐	☒	Estimates of effect sizes (e.g. Cohen's *d*, Pearson's *r*), indicating how they were calculated

Our web collection on statistics for biologists contains articles on many of the points above.

Software and code

Policy information about availability of computer code

Data collection	Illumina HiSeq 2000 to generate bulk RNA-seq data. Illumina NovaSeq 6000 to generate scRNA-seq data.
Data analysis	Raw gene expression matrices generated per sample using CellRanger (https://support.10xgenomics.com/single-cell-gene-expression/software/pipelines/latest/installation, version 4.0.0) were further anlysezed in R (version 3.6.3). Matrices were converted and combined to a Seurat object using the Seurat (R package , version 3.2.2). To identify cell doublets, Scrublet (Python package, version 0.2.2) and DoubletDecon (R package, version 1.1.6) were used for inference. In the descriptions of cell subtypes, SingleR (R package, version 1.0.6), GSVA (R package, version 1.34), Metascape (https://metascape.org/gp/index.html), Monocle2 (R package, version 2.14), pySCENIC (Python package, version 0.9.9) were comprehensively used. In addition, malignant tumor cells were distinguished in epithelial cells with help of inferCNV (R package, version 1.2.1), and then five biological gene modules were identified using the NMF (R package, version 0.23) followed by related analyses. CellPhoneDB (https://github.com/Teichlab/cellphonedb, version 2.0.6) used the cluster annotations and raw counts from our single-cell transcriptomics data to compute cell-cell communication within the cell subtypes.<br>For bulk RNA-seq data of 56 clinical samples (44 samples with 12 additional ones), pair-end reads were aligned to the human genome (GRCh38) using HISAT2 (https://github.com/DaehwanKimLab/hisat2, version 2.1.0). Software featureCounts (part of Subread package, http://subread.sourceforge.net/, version 2.0.3) was used to quantitate the read counts of each gene in samples, and then the expression levels of genes were normalized by gene length and sequencing depth with edgeR (R package, version 3.28.1) among samples.<br>CIBERSORTx (https://cibersortx.stanford.edu/, version 1.1.0) deconvoluted bulk RNA-seq data including both of our HPC and public NPC cohorts into the subtype compositions in each sample using the S-mode batch correction.<br>Custom codes developed in the study are available at https://github.com/Sara0201Tao/2022HPC.

For manuscripts utilizing custom algorithms or software that are central to the research but not yet described in published literature, software must be made available to editors and reviewers. We strongly encourage code deposition in a community repository (e.g. GitHub). See the Nature Portfolio guidelines for submitting code & software for further information.

Data

Policy information about availability of data

All manuscripts must include a data availability statement. This statement should provide the following information, where applicable:

- Accession codes, unique identifiers, or web links for publicly available datasets
- A description of any restrictions on data availability
- For clinical datasets or third party data, please ensure that the statement adheres to our policy

The raw sequence data generated from bulk and single-cell RNA-seq of clinical samples in this study have been deposited in the Genome Sequence Archive (Genomics, Proteomics & Bioinformatics 2021) in National Genomics Data Center (Nucleic Acids Res 2022), China National Center for Bioinformation / Beijing Institute of Genomics, Chinese Academy of Sciences, under accession number HRA003383 [https://ngdc.cncb.ac.cn/gsa-human/browse/HRA003383]. The data are available under restricted access for relevant data protection regulations considering the data contain human genetic information, and the access can be obtained after being authorized by its Data Access Committee (DAC) by checking the identity and purpose of applicants. Generally, reasonable requests will be approved within 2 weeks and the download permission will be opened.
The publicly available NPC bulk RNA sequencing data used in the study are available in GEO with the accession number GSE102349 [https://www.ncbi.nlm.nih.gov/geo/query/acc.cgi?&acc=GSE102349]22.
The patient information for single-cell sequencing and bulk RNA sequencing is available in Supplementary Tables 1, 2, 3 and 5. Source data are provided with this paper.

Human research participants

Policy information about studies involving human research participants and Sex and Gender in Research.

Reporting on sex and gender	According to the 2020 statistics form cancer today (https://gco.iarc.fr/today/home) and relevant research (DOI: 10.1159/000492299), there is a significant gender difference in incidence of hypopharyngeal carcinoma (HPC) worldwide, with especially more 10-fold difference in late life at the ratio of 5.1: 0.4 in Taiwan and 1.17:0.04 in Korea between men and women. All these information indicate that HPC is a male predominate cancer with relatively rare female patients. This is mainly because smoking and alcohol abuse are the main causes of hypopharyngeal carcinoma in addition to HPC virus infection.<br>Therefore, in the recruitment process, we did not enroll patients based on their gender information. According to Supplementary Table 1, 2 and 5, the majority of patients we recruited were also male.
Population characteristics	Totally 64 patients with pathological HPC diagnosis were enrolled in this study. None of the patients had received prior treatment. Detailed information can be found in the Patient recruitment and sample collection section of Methods and Supplementary Table 1, 2 and 5. In summary, patients are 43-73 years old males and females, most of who had a history of smoking and drinking and were diagnosed as different clinical stages of T2-T4.
Recruitment	All donors are recruited and managed by Department of Otolaryngology Head and Neck Surgery in Beijing Tongren Hospital, avoiding the selection of poorly clinically characterized volunteers. Eight patients who were radiologically and pathologically diagnosed with advanced hypopharyngeal carcinoma (HPC) were enrolled in this study between December 2019 and January 2021 for scRNA-seq profiles. Additionally, 44 HPC samples of our HPC cohort and another 12 HPC samples for respective research were collected from 56 individual patients and sent for bulk RNA-seq between July 2016 and August 2022.
Ethics oversight	Written informed consent was obtained from all above 64 participants, and ethical approval was obtained from the Ethics Committee of Beijing Tongren Hospital, Capital Medical University (TRECKY2016-025 and TRECKY2021-049).

Note that full information on the approval of the study protocol must also be provided in the manuscript.

Field-specific reporting

Please select the one below that is the best fit for your research. If you are not sure, read the appropriate sections before making your selection.

☒ Life sciences ☐ Behavioural & social sciences ☐ Ecological, evolutionary & environmental sciences

For a reference copy of the document with all sections, see nature.com/documents/nr-reporting-summary-flat.pdf

Life sciences study design

All studies must disclose on these points even when the disclosure is negative.

Sample size	The HPC is a rare malignancy. Sample size for 10x Genomics scRNA-seq was determined by the availability of patient samples. No statistical tests were performed for sample size calculation but it was sufficient for this proof-of-concept study. The exact number of samples used per figure is informed in each figure. In the whole manuscript, we used 15 samples for single-cell RNA-seq analysis and 56 samples for bulk RNA-seq analysis.
Data exclusions	All criteria for data exclusion were pre-established. Quality filtering was firstly performed to remove cells with < 201 or > 7500 expressed

Data exclusions	genes, cells with more than 25% unique molecular identifiers (UMIs) derived from the mitochondrial genome. For further and detailed quality control of cells, we used package Scrublet and DoubletDecon to infer cell doublets, and checked with feature gene expressions to remove unqualified events including doublets, contaminating cells and cells with high fraction expression of dissociation genes.
Replication	Tumor is heterogeneous. We used bulk RNA-seq data to verify the findings of single-cell RNA-seq data.
Randomization	The HPC patients were recruited randomly in this study. And the divided groups were determined according to clinical diagnosis.
Blinding	Investigators were blinded to patient identity. Tumour biopsy was collected and sent for downstream procedures with coded Sample_ID.

Reporting for specific materials, systems and methods

We require information from authors about some types of materials, experimental systems and methods used in many studies. Here, indicate whether each material, system or method listed is relevant to your study. If you are not sure if a list item applies to your research, read the appropriate section before selecting a response.

Materials & experimental systems

n/a	Involved in the study	
☐	☒	Antibodies
☒	☐	Eukaryotic cell lines
☒	☐	Palaeontology and archaeology
☒	☐	Animals and other organisms
☒	☐	Clinical data
☒	☐	Dual use research of concern

Methods

n/a	Involved in the study	
☒	☐	ChIP-seq
☒	☐	Flow cytometry
☒	☐	MRI-based neuroimaging

Antibodies

Antibodies used	Four primary antibodies are used in our study after dilution as follows: anti-EPCAM (rabbit; Abcam; Cat. no. ab223582; 1/100), anti-FAP (rabbit; Abcam; Cat. no. ab207178; 1/100), anti-CD31 (mouse; CST; Cat. no. CST3528; 1/300), anti-CD45 (rabbit; CST; Cat. no. CST13917; 1/400). In addition, polymer HRP Ms+Rb has been used as the secondary antibody (GT no. GK600711-B).
Validation	All the antibodies used in this study were commercial antibodies, with validation procedures described on the following sites of the manufacturers:<br>anti-EPCAM (rabbit; Abcam; Cat. no. ab223582)<br>https://www.abcam.com/epcam-antibody-epr20532-225-ab223582.html<br>anti-FAP (rabbit; Abcam; Cat. no. ab207178)<br>https://www.abcam.com/fibroblast-activation-protein-alpha-antibody-epr20021-ab207178.html<br>anti-CD31 (mouse; CST; Cat. no. CST3528)<br>https://www.cellsignal.cn/products/primary-antibodies/cd31-pecam-1-89c2-mouse-mab/3528<br>anti-CD45 (rabbit; CST; Cat. no. CST13917)<br>https://www.cellsignal.cn/products/primary-antibodies/cd45-intracellular-domain-d9m8i-xp-rabbit-mab/13917?site-search-type=Products&N=4294956287&Ntt=13917&fromPage=plp&_requestid=426012<br>polymer HRP Ms+Rb (GT no. GK600711-B)<br>https://www.genetech.com.cn/goods/goods_detail/757861.html

March 2021
